# Research productivity grants: Physical Education, Physical Therapy, Speech Pathology, and Occupational Therapy

**DOI:** 10.1590/bjpt-rbf.2014.0150

**Published:** 2016

**Authors:** Brasília M. Chiari, Débora B. Grossi, Fernanda D. Fernandes, Leslie P. Ferreira, Marco T. Mello, Pedro C. Hallal, Sérgio T. Fonseca

**Affiliations:** 1 Universidade Federal de São Paulo (UNIFESP), São Paulo, SP, Brazil; 2 Universidade de São Paulo (USP), Ribeirão Preto, SP, Brazil; 3 Pontifícia Universidade Católica de São Paulo (PUC), São Paulo, SP, Brazil; 4 Universidade Federal de Minas Gerais (UFMG), Belo Horizonte, MG, Brazil; 5 Universidade Federal de Pelotas (UFPel), Pelotas, RS, Brazil

The research productivity grants (PQ) distributed annually by Conselho Nacional de Desenvolvimento Científico e Tecnológico - CNPq (National Council for Scientific and Technological Development) have generated a number of concerns in the scientific community. It is the purpose of this editorial to shed light on the criteria adopted by the Multidisciplinary Health Advisory Committee (AC) for granting PQ grants.

A key point is to emphasize that the PQ grants of this AC are distributed primarily to researchers with initial training in the areas of the committee and with institutional ties to units, departments, or graduate programs of these areas. Supervision of candidates in programs that are not accredited by Coordenação de Aperfeiçoamento de Pessoal de Ensino Superior - CAPES (Coordination for the Improvement of Higher Education Personnel) are ineligible, as are scientific works not related to the areas of the committee.

It should be noted that there are minimum entry criteria for each level, which are publicly available on the CNPq website. Regrettably, the AC continues to receive numerous applications that do not meet those criteria, including applications from undergraduate or graduate students.

A diagnosis of the situation of the AC is in order at this point. Physical Education currently has 85 existing grants, Physical Therapy and Occupational Therapy have 65 grants and Speech Pathology has 51 PQ grants. The Physical Education grants are distributed among eight states of the Federation, with 40% allocated to teachers in the state of São Paulo. The Physical Therapy and Occupational Therapy grants are allocated to researchers in seven states, with 63% of grant holders working in the state of São Paulo. In Speech Pathology, the grants are concentrated in six states, with 71% allocated to researchers with ties to institutions in São Paulo.

Of the total AC grants, 62.5% are level 2, 18.5% are level 1D, 5.5% are 1C, 8.5% are 1B, and 5.0% are 1A. These percentages are at odds with CNPq standards, which recommend 10% of grants in levels 1A and 1B and 50% at level 2. The AC has forwarded documents to the senior management at CNPq requesting a review of this scenario and an increase in level 1 grants, particularly 1A and 1C. It is also worth noting that, in the last three appraisals, there was no allocation of new grants for our AC. The matter was also discussed in a recent document forwarded to senior management at CNPq.

We will now present some information about the appraisal of PQ grants in 2015. The most complex task of the assessment was to define the indicators to be included in the calculation algorithm, with respective weights. The AC has chosen to use five indicators, with the following weights: scientific production during the study period (35%), supervision (25%), H index from the Institute of Scientic Information - ISI (20%), average number of citations per paper based on data from the Scopus database (15%), and submitted research project (5%). It should be noted that these criteria were used only for the applicants who met the minimum criteria published in the CNPq website. For example, researchers with predominant activity in other areas or who did not reach the minimum criteria of supervision and scientific production were eliminated at this first stage of the assessment.

Each of these indicators has a specific algorithm with standardized calculation, and the final score ranges from 0 to 100. For the project, they are evaluated by two ad-hoc reviewers. An "excellent" rating is equivalent to a score of 5, a "good" rating is equivalent to a score of 3, a "fair" rating is equivalent to a score of 2, and a "poor" rating is equivalent to a score of 1. The average of the two assessments is used in the final score. In the 2015 appraisal, only 16% of the projects were rated "excellent" by both evaluators.

Regarding the H index, the 95th percentile of the distribution is calculated and this value is equivalent to the maximum score (20). The score of each researcher is determined by using a rule-of-three calculation compared to the value of the 95th percentile. In gross terms, the average H index of applicants in 2015 was 5.2, ranging from 0 to 19. The 95th percentile corresponded to 13. The average H index of the Physical Education applicants was 5.4, compared to 6.0 in Physical Therapy and Occupational Therapy, and 3.0 in Speech Pathology.

Regarding citations, the calculation is made in exactly the manner that the H index calculation is made. In gross terms, the average number of citations per paper was 5.6, with an average of 5.2 citations/paper in Physical Education, 6.6 in Physical Therapy and Occupational Therapy, and 4.0 in Speech Pathology.

The calculation of the scientific production score is complex. Each article published in journals with impact factor greater than four equals 100 points. The weight of the publications in journals with lower impact decreases gradually, reaching 50 for publications in journals with impact factor between 0 and 0.5 and 10 for publications in journals without impact factor. Articles published in journals without peer review are disregarded. Each book is equivalent to 80 points and each book chapter is equivalent to 40 points. Bonus points are awarded for publications as first or last author, and then as a second or second-to-last author. This algorithm generates a continuous score, which is then processed in the same manner as described for the H index and citation index.

In the Guidelines, three points are awarded for completed doctoral supervisions, two points for completed postdoctoral supervisions, and one point for completed master's supervisions. Half of these values ​​is assigned to supervisions in progress. The final score is calculated with a weight of 25 in a manner similar to that performed for the other indicators. However, this indicator has a ceiling value, equivalent to the title of 0.5 doctor and 1 master per year. All lecturers who reach these values ​​receive the maximum score in the supervision category.

The continuous score, with a weight of 100, correlates with all indicators. The strongest correlation was with the H index (r = 0.83), followed by scientific production (r = 0.73), average number of citations (r = 0.66), supervisions (r = 0.58), and research project (r = 0.42). The final average score was 47 points for Physical Education applicants, 48 for Physical Therapy and Occupational Therapy applicants, and 36 for Speech Pathology applicants.

Given the availability of the grant renewals in 2015, it was possible to meet 30% of the demand for Physical Education, 31% of the demand for Physical Therapy and Occupational Therapy, and 49% of the demand for Speech Therapy. These figures demonstrate the competitiveness of the system and encourage this AC to seek the constant improvement of the evaluation criteria.

The AC then carried out a number of analyses comparing the researchers who received grants to those who did not in 2015. In Physical Education, the average H index for grant recipients was 8.3, compared to 2.0 for non-recipients. The average number of citations was 8.2 for recipients and 2.7 for non-recipients. In Physio Therapy and Occupational Therapy, the differences were smaller. The average H index was 7.6 for recipients, compared to 4.8 for non-recipients. The average numbers of citations were 11.1 and 4.1, respectively. In Speech Pathology, the average H index was 3.6 for recipients, compared to 1.8 for non-recipients. The average number of citations was 4.6 for recipients and 2.0 for non-recipients.

The AC would like to take this opportunity to raise some issues related to the area. Lattes résumés need to be written appropriately. We have detected many instances of conference abstracts included as "Full Papers Published in Journals" in the applicants' résumés. Similarly, editorials and letters to the editor are not always clearly identified as such by the applicants. There is an increasing tendency by the committee to place more emphasis on the quality of the scientific production than on mere quantity. At the 2014 appraisal, production had a weight of 50 points, however this was educed to 35 points in 2015. The H index doubled in value and the weights of the citation index and supervision history have increased.

We would also like to take this opportunity to thank the ad hoc reviewers of the AC. However, some considerations should be made. The reviews are often too succinct, preventing a more adequate understanding of the submitted projects. It should also be noted that the overall assessment of the ad-hoc review for the PQ grants should focus on the submitted project, not on the applicant's résumé, which is assessed according to the other indicators described herein. Finally, it is essential that the reviewers assess the proposals based on the criteria established by the AC.

In conclusion, the AC promises to publish the detailed results of all appraisals conducted, within the legal restrictions imposed by CNPq and by the Federal Constitution. Applicants are welcome to contact the members of the AC via email regarding any questions about the appraisal or its general and specific scores for each indicator.

This editorial is being published simultaneously in the Brazilian Journal of Physical Therapy, Cadernos de Terapia Ocupacional da UFSCar, CoDAS, and Movimento, as well as the CNPq website. The purpose of this joint publication is to reach researchers from the different areas that make up the AC. We hope that it will generate a broad scientific discussion about constant improvement of the AC's evaluation criteria.

